# Reactive Astrogliosis: Implications in Spinal Cord Injury Progression and Therapy

**DOI:** 10.1155/2020/9494352

**Published:** 2020-08-19

**Authors:** Xinyu Li, Meng Li, Lige Tian, Jianan Chen, Ronghan Liu, Bin Ning

**Affiliations:** Jinan Central Hospital, Cheeloo College of Medicine, Shandong University, No. 105, Jiefang Road, Jinan, Shandong 250013, China

## Abstract

Astrocytes are the most populous glial cells in the central nervous system (CNS). They are essential to CNS physiology and play important roles in the maintenance of homeostasis, development of synaptic plasticity, and neuroprotection. Nevertheless, under the influence of certain factors, astrocytes may also exert detrimental effects through a process of reactive astrogliosis. Previous studies have shown that astrocytes have more than one type of polarization. Two types have been extensively researched. One is a damaging change that occurs under inflammation and has been termed A1 astrocyte, while the other is a restorative change that occurs under ischemic induction and was termed A2 astrocyte. Researchers are now increasingly paying attention to the role of astrocytes in spinal cord injury (SCI), degenerative diseases, chronic pain, neurological tumors, and other CNS disorders. In this review, we discuss (a) the characteristics of polarized astrocytes, (b) the relationship between astrocyte polarization and SCI, and (c) new implications of reactive astrogliosis for future SCI therapies.

## 1. Introduction

Astrocytes are the most numerous cells in the mammalian central nervous system (CNS) and are responsible for various functional and structural roles [1]. Astrocytes regulate blood flow [2, 3], provide energy metabolites to neurons [4], participate in synaptic function and plasticity [1], maintain the balance of extracellular ions and fluids [2], and control neurotransmitter production [5]. The roles of astrocytes in various pathological processes and injuries have been increasingly acknowledged in recent literature [1]. Astrocytes respond to numerous forms of CNS insults through a process referred to as reactive astrogliosis, which has become a pathological hallmark of CNS lesions. For example, reactive astrocytes have been considered a pathological hallmark of many neurodegenerative diseases, including Parkinson's and Alzheimer's diseases, as well as amyotrophic lateral sclerosis [6]. Some reports have introduced the role of reactive astrocytes in chronic pain [7]. In 2019, Henrik Heiland et al. reported a type of tumor-associated reactive astrocyte, which aids the progression of glioblastoma through the JAK/STAT pathway [8]. Even in the normal aging brain, reactive astrocytes have been found in certain brain regions [9]. In recent years, researchers have gradually recognized various effects of reactive astrocytes in the process of SCI recovery, including a decrease in the infiltration of inflammatory cells after injury [10]; secretion of related neurotrophic factors [11]; secretion of a significant amount of extracellular matrix, leading to limited nerve repair [12]; and failure to form glial scars, leading to axonal degeneration [13, 14]. Considering the specific process of astrocyte polarization in a wide range of diseases [15, 16], further understanding can shed light on potential directions for future astrocyte therapy. Therefore, in this review, we focus on the development of astrocyte polarization and its relationship with SCI.

## 2. Astrocyte Reactivity

### 2.1. Development of the Concept of Astrocyte and Astrocyte Reactivity

The concept of astrocyte was first proposed by Virchow, who described the astrocyte as a kind of cell that surrounds neurons and provides them with nutrients [17]. After the advancement of microscopy and dyeing techniques, Otto Dieters was the first to visualize astrocytes and provide key descriptions, while Camillo Golgi was the first to prove that astrocytes differ from neurons; however, he also believed that astrocytes and neurons could be transformed from one to the other. Lenhossék introduced the term “astrocyte” in 1895 [18] and developed astrocyte-specific gold and mercury chloride-sublimation staining techniques that label glial fibrillary acidic protein (GFAP); it was this staining that allowed Cajal to discriminate astrocytes from radial glial cells [19]. GFAP and vimentin constitute intermediate filaments, and these components form the basic skeleton of an astrocyte [1]. At the time, GFAP was used as a standard marker for astrocyte reactivity. It is important to mention that GFAP is reportedly expressed in all mammals, but not in fish [20, 21], amphibians [22], turtles [23], and some other nonmammals [16]. Although GFAP may not be the most accurate marker of astrocyte activity, it provides a convenient method of detection. Due to advances in methods for isolating astrocytes and improved understanding of the genetic changes in reactive astrocytes, some new hallmarks of reactive astrocytes have been confirmed, such as Lcn2 and SerpinA3N [24–26]. Interestingly, the concept of modern astrocyte polarization was also proposed by Virchow, who gave a comprehensive description of astrocyte polarization after trauma and neurodegenerative injury [27]. In 2012, Zamanian et al. proposed methods for the formation of two types of reactive astrocytes. The authors elicited the neuroinflammation model by systemic LPS injection and generated an ischemic model by middle cerebral artery occlusion. Using these models, they found two different types of astrocytes that are called A1 and A2 astrocytes, which are similar to the M1/M2 macrophage paradigm [16, 26]. It should be noted that the current naming rules are remarkably simple and that A1 and A2 are not the only types of reactive astrocytes. The naming convention of A1/A2 is used to facilitate scientific research and communication. The function of A1 astrocytes is greatly reduced; for example, there is a lower number of A1 astrocyte-induced synapse formations and the function of these synapses is insufficient. A1 astrocytes highly upregulate the expression of several classical complement cascade genes that were previously shown to be destructive to synapses. Moreover, A1 astrocytes secrete a soluble toxin that rapidly kills a subset of CNS neurons and mature oligodendrocytes but does not affect other CNS cell types [16, 26]. Thus, A1 astrocytes are considered to be a potentially harmful type. In contrast, A2 astrocytes have been shown to upregulate several neurotrophic factors and thus may be a protective type. A2 astrocytes promote the expression of anti-inflammatory cytokine transforming growth factor *β* (TGF-*β*) [28]. This factor contributes to axon formation and neuroprotection [29]. Complement component 3 has been found to be specifically elevated in A1 astrocytes, and it is not expressed in A2 astrocytes; thus, it is an effective new marker for detecting A1 astrocytes. S100A10 is a member of the S100 protein family and has been shown to be a specific hallmark for A2 astrocytes. Additionally, S100A10 plays roles in cell proliferation, membrane repair, and inhibition of apoptosis [30].

### 2.2. Characteristics of Reactive Astrogliosis

In order to determine the roles of astrocytes in the pathology and disorders of the CNS, a large number of recent studies have investigated the functions and mechanisms of reactive astrogliosis. Reactive astrogliosis is not a single stereotypic reaction; in contrast, it is a series carefully regulated changes, including gene expression, cellular hypertrophy, scar formation, and long-term organizational changes [31]. Additionally, reactive astrogliosis is not an all-or-nothing phenomenon, but rather a progressive phenomenon that occurs in a context-dependent manner and that is regulated by specific signaling events. When the initial stimulus to induce astrogliosis is relatively mild, the reactive astrocytes have the ability to return to a relatively normal state after removing the triggering environment [32]. The changes in astrocyte polarization vary with the intensity and type of stimulation. Changes in molecular expression occur at the initial stage, and as the intensity of the stimulus increases, cell hypertrophy, proliferation, and scar formation take place. In a previous study [26], GFAP-stained cerebral cortical mouse cells were used to stimulate the cells by injecting lipopolysaccharides (LPS). As intensity of the stimuli increased, astrocytes overlapped and scars were eventually formed at the site of stimulation. During the polarization of astrocytes, there is a rapid transformation of gene expression. Interestingly, despite the persistent expression of some core genes, at least 50% of the altered gene expression strongly depends on the type of injury [26]. Reactive astrogliosis consists of gene expression and cellular changes that are controlled by complex intercellular and intracellular signals. Currently, it is believed that the most important cellular type that triggers reactive astrogliosis is microglia. In 2017, Liddelow et al. reported that A1 astrocytes are induced by activated microglia [33]. The authors found that three factors, Il-1*α*, TNF*α*, and C1q, are produced by microglia and cause astrocyte polarization and that these three factors are essential for LPS-induced in vitro polarization. A single gene knockout test showed that these three factors work together to induce the formation of A1 astrocytes. Microglia do not induce astrocyte polarization when only one or two genes are present. Sonn et al. found that IL-4-induced M2 macrophages could stimulate astrocyte activity and chemotaxis of reactive astrocytes in SCI through the Wnt/*β*-catenin pathway. They observed that the percentage of elongated reactive astrocytes significantly decreased, and the orientation of elongated astrocytes was more random, when the Wnt/*β*-catenin pathway was inhibited. In contrast, the Wnt pathway agonists enhanced the average protrusion length compared to a control group [34]. Further exploration to determine whether IL-4–induced M2 microglia have the same function will be helpful.

### 2.3. Morphological Changes in Reactive Astrocytes

**Figure 1:**
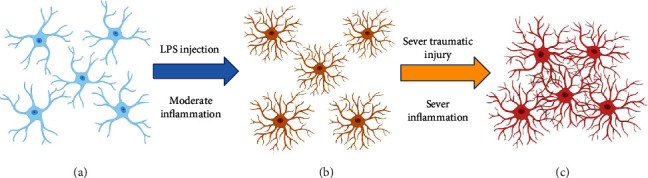
As the degree of stimulation deepens, reactive astrocytes will also undergo different morphological changes. (a) Astrocytes under normal physiological conditions. (b) When the injury is moderate, the processes of reactive astrocytes increase, accompanied by hypertrophy of the process and cell body. (c) Reactive astrocyte further hypertrophy and overlap.

Astrocyte polarization induces morphological changes and striking increases in GFAP immunoreactivity. These findings have been interpreted as cellular hypertrophy [35]. It is currently believed that the morphological changes that occur in reactive astrocytes are mainly the characteristic hypertrophy of the cell body and processes. In addition, the number of protrusions and the length will increase. The morphological changes of reactive astrocytes will vary depending on the type, intensity, and location of the astrocytes at the time of injury (Figure 1). Labeling with GFAP shows that the thickness of processes varies in different types of injuries. For example, the process thickness in cortical and hippocampal injuries increased 2-fold [36]. In an optic nerve crush model, a 0.5-fold increase was observed in the protrusion thickness [37]. However, the thickness of the process was elevated 2.5-fold in an epilepsy injury [38]. Reactive astrocytes exhibit different morphologies as the distance from the lesion core varies. Reactive astrocytes exhibit a typical hypertrophic process and stellate shape without preferential orientation of the protrusion when they are far from the lesion core. However, reactive astrocytes adjacent to the lesion core show a longer process that is oriented towards the damaged core [34].

### 2.4. Functional Changes of Reactive Astrocytes

At present, accumulating evidence suggests that astrocytes lose their normal physiological functions and gain various abnormal functions during the process of reactive astrogliosis [5, 39, 40]. For example, in Alzheimer's disease, astrocytes become reactive due to the deposition of amyloid *β*-protein, and the resulting detrimental consequences include impaired glutamate uptake owing to decreased expression of uptake transporters, altered energy metabolism, altered ion homeostasis (K+ and Ca+), impaired synaptic plasticity, and increased release of cytokines and chemokines [41–43]. Using transgenic mice, which selectively express *α*-synuclein (a hallmark of Parkinson's disease) in astrocytes, Gu et al. have shown impaired maintenance of the blood-brain barrier (BBB) and decreased excitatory amino acid transporters of reactive astrocytes [44]. In addition, reactive astrocytes undergo a series of abnormal changes in signaling pathways, such as impaired lactate transport [45], downregulated GLT-1 expression, and sustained calcium ion release in amyotrophic lateral syndrome (ALS). Similarly, reactive astrocytes in ALS also exhibit a malabsorption of glutamate, which is toxic to neurons [6].

Initially, researchers paid greater attention to the detrimental effects of reactive astrocytes in various diseases. Thus, the general belief was that inhibiting the formation of reactive astrocytes would completely block its negative effects. However, as the evidence grows, this simple view no longer remains tenable. Many experiments have shown that reactive astrocytes have positive roles [32]. For example, in GFAP and Vim KO mice, glial scar formation was impaired and less dense scars (frequently accompanied by bleeding) were formed [25, 26]. In 2013, Kraft et al. showed that reactive astrocytes can limit plaque formation in Alzheimer's disease and reduce plaque-associated dystrophic neurite formation [27]. In cerebral ischemic injury, reactive astrocytes can play a protective role while regulating glutamate transport and control of gap junctions. Specific deletion of reactive astrocytes will increase the degree of damage and neuronal lesions [46].

Although reactive astrocytes do have some positive effects, the abnormal functions of them are more notable. At present, the negative effects of reactive astrocytes mainly include the following: (a) production of proinflammatory chemokines and cytokines [26, 47, 48]; (b) generation of Ca2+ signals [49, 50]; (c) upregulation of aquaporin 4 (AQP4), leading to cytotoxic edema in spinal cord injury and ischemia [51]; (d) elevation of neuroexcitatory glutamate levels [52, 53]; (e) promotion of proliferation and migration or formation of an immunosuppressive environment in glioblastoma [8, 54]; (f) contribution to induction and maintenance of chronic pain [7]; and (g) maintenance and promotion of neurodegenerative diseases [6]. Due to the context-dependent nature of reactive astrocytes, descriptions of their role from different reports of the same disease model may also be different, or they may even be reported to have opposite regulatory effects. Therefore, the function of reactive astrocytes cannot be easily determined, and the pros and cons of reactive astrocytes should be comprehensively evaluated by considering the type of injury, the degree of stimulation, the location of the lesion, and the time after the injury.

### 2.5. Signaling Pathways and Molecules Triggering Reactive Astrogliosis

**Figure 2:**
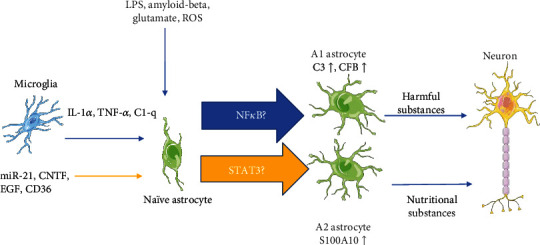
At present, due to the similarity between the NF*κ*B pathway and some of the functions mediated by A1 astrocytes as well as similarities between the STAT3 pathway and some of the actions of A2 astrocytes, it may be that astrocytes transform into A1 through the NF*κ*B pathway and into A2 through the STAT3 pathway.C3: complement component 3; CFB: complement factor B; S100A10: S100 calcium-binding protein A10.

Many signaling pathways are related to astrocyte reactivity [55–57]. We summarized these molecules and pathways in Table 1. The most important of these are the NF*κ*B and STAT3 pathways. There is growing evidence that activation of the NF*κ*B pathway leads to pathological changes in the CNS and that inhibition of this pathway helps to limit disease progression [47, 58, 59]. One study demonstrated that selective inactivation of the astroglial NF*κ*B pathway in transgenic mice led to an enormous improvement in the function of the spinal cord after SCI, while also resulting in a decreased trend of proinflammatory chemokines and cytokines, such as CXCL10, CCL2, and TGF-*β*2 [47]. Transgenic inactivation of astroglial NF*κ*B alleviated disease severity and elevated functional recovery after experimental autoimmune encephalomyelitis (EAE) [60]. The STAT3 pathway plays an important role in causing polarization of astrocytes, including in both acute injury and neurodegenerative diseases [61]. Activation of the STAT3 pathway has many functions that are opposed to the NF*κ*B pathway, and it generally contributes to damage repair and cell survival. The STAT3 pathway also plays an important role in scar formation. Conditional knock-out of STAT3 from astrocytes will lead to the downregulation of GFAP, failure of astrocyte hypertrophy, and apparent failure of astrocyte scar formation after SCI in addition to the diffusion of inflammation and impaired motor recovery [62]. Okada et al. revealed the important role of the STAT3 pathway in injury repair. By using mice with a selective deletion of the protein signal (STAT3) or the protein suppressor of cytokine signaling 3 (SOCS3) under the control of the promoter-enhancer, they found that when the STAT3 pathway was inhibited, it caused significant inflammatory cell infiltration, neurological disorders, demyelination, and severe neurological dysfunction. On the contrary, in the activation of the STAT3 pathway, they found that reactive astrocytes migrated rapidly to the lesion and blocked inflammatory cells, increased contraction of tissue in the lesion area, and improved function recovery. These results suggest that STAT3 is a key regulator of reactive astrocytes in the healing process after SCI, thus providing a potential intervention target in the treatment of SCI [10]. In summary, it is probably that reactive astrocytes cause pathological damage through the NF*κ*B pathway and lesion repair and inflammation recovery through the STAT3 pathway (Figure 2). TGF-*β* signaling is considered another important factor of reactive astrogliosis in SCI. TGF-*β* signaling has been found to be a key upstream trigger of CSPG expression in glial scar formation [63, 64]. The blood protein fibrinogen, which leaks into the CNS after blood-brain barrier disruption or vascular damage, serves as an important factor for the glial scar formation via the TGF-*β* signaling pathway. Further, it has also been found that genetic or pharmacologic depletion of fibrinogen in mice reduces TGF-*β* signaling pathway-activated reactive astrogliosis after SCI. Moreover, injection of fibrinogen into the mouse cortex is sufficient for inducing RAs. In vivo and in vitro inhibition of the TGF-*β* pathway blocks the function of fibrinogen in the glial scar formation [64]. It is worth mentioning that our team has found that miR-21 regulates reactive astrogliosis by TGF-*β* signaling at the postacute phase of SCI [65]. More recently, we demonstrated that miR-21 is a switch that regulates the polarization of astrocytes. When miR-21 is downregulated, A1 astrocytes transform into A2 astrocytes. Conversely, when miR-21 is upregulated, A2 astrocytes transform into A1 astrocytes. Furthermore, this function is mediated through the STAT3 pathway. A2 astrocytes regulated by miR-21 promoted synapse formation and neurite growth by targeting Gpc6 and GDNF though the STAT3 signaling pathway [66]. The epidermal growth factor receptor (EGFR) has been found to be a mediator of reactive astrocytes. It is a transmembrane receptor tyrosine kinase that regulates basic cellular functions. After SCI, the EGFR is upregulated primarily in astrocytes at the site of injury and triggers astrocyte polarization [67, 68]. EGFR activation mediates the secretion of growth inhibitory molecules at the site of injury, which can aggravate the formation of glial scars. It has also been shown that treatment with EGFR-specific inhibitors can reduce the formation of glial scars and improve the prognosis. Nucleolin has been shown to interact with epidermal growth factors and to cause an increase in its activation. The use of the nucleolin inhibitor can reduce the activation of EGFR, the proliferation of astrocytes, and the secretion of CSPGs, thereby improving the prognosis of SCI [69].

### 2.6. Genetic and Molecular Changes in Reactive Astrocytes

Previous studies have shown that reactive astrocytes produce many types of intercellular signaling molecules, including cytokines, chemokines, energy metabolism molecules, toxic amino acids, or intracellular signaling molecules including ions and receptors through gene expression (Table 2). These molecules affect themselves and surrounding cells, such as nerve cells, oligodendrocyte precursor cells, and microglia. The expression of genes and molecules of reactive astrocytes is very complicated, and the changes of genes and molecules in different environments also differ. Under the same stimulation, reactive astrocytes in different tissue parts will induce different gene expressions. For example, in different parts of mice with traumatic spinal cord injury, reactive astrocytes were found to express different levels of brain lipid-binding protein (BLBP), GFAP, and nestin [81]. This demonstrates that the astrocyte response to SCI is regionally heterogeneous. Furthermore, only certain types of astrocytes will polarize or be proliferative during the lesion of cortical stab injury [82]. Most of these proliferative cells are located in the juxtavascular sites. Using clonal analysis, the proliferative reactive astrocytes were found to come from different progenitor cells, indicating that the specificity of astrocytes may be decided by distinct cellular origin [83, 84]. This may be related to the birth fate decision of astrocytes, which means that the astrocytes from different sources were carved with their own characteristic at the moment they were produced. Thus, they have slightly different functions, and different gene expressions will also be produced during the polarization of astrocytes. Due to the specificity of the individual cells, whole-transcriptome gene sequencing for population gene expression does not seem to be a good solution to this problem. Single-cell RNA sequencing is a promising method for discriminating the characteristics of different reactive astrocytes.

## 3. The Roles of Astrocyte Polarization in SCI

### 3.1. Introduction to Spinal Cord Injury

SCI is a condition associated with permanent physical disability that requires long-term and complex health care [104]. According to reports, there are 5,000 new patients with traumatic SCI per year in Japan and approximately 17,000 new patients per year in the United States [104, 105]. Etiologically, 90% of cases are caused by violence and accidents, including sport accidents and falls. Clinical manifestations depend on the severity and location of the injury, and the analysis of performance considers partial or complete loss of sensorimotor activity. SCI has a devastating impact on the family of patients, as it prevents them from being economically active. However, despite research on SCI for many years, there is still no effective way to completely restore function of the damaged spinal cord, and more research is needed to understand the mechanisms that reduce inflammation at the injury site and restore spinal cord regeneration. The process of SCI mainly includes primary injury and secondary injury. Primary injury refers to a mechanical SCI caused by vertebral fracture or dislocation. Secondary injury generally occurs within a few minutes after the primary injury and lasts for several weeks or months, causing continuous damage to the spinal cord [106]. Secondary injury can be temporally divided into acute, subacute, and chronic phases. The acute phase occurs quickly after injury and includes astrocyte polarization, vascular damage, ion imbalance, neurotransmitter accumulation, free radical formation, and inflammation [106, 107]. The subacute stage includes apoptosis, demyelination of surviving axons, and matrix remodeling. Finally, the chronic stage includes scar formation and failure of axonal connections [106, 108].

### 3.2. Detrimental Function of Astrocyte Activity in SCI

#### 3.2.1. Effect of Reactive Astrocytes on Axon Regeneration

The detrimental effects of reactive astrocytes in axonal regeneration mainly include formation of a physical barrier to obstruct axon growth and secretion of inhibitory proteins that impede functional recovery. During the chronic phase of SCI, astrocytes transform into scar-forming astrocytes [109, 110]. Scar-forming astrocytes, microglia, and NG2 glia together form a dense boundary structure around the lesion core to isolate the entire damaged area. After the inflammatory response has stabilized, there are still some other cellular components in the lesion core, including pericytes, perivascular-derived fibroblasts, ependymal cells, and peripheral-derived immune cells [111]. The above components together constitute glial scars. Glial scars are thought to be a mechanical barrier to nerve fiber regeneration and primordial cell regeneration [14]. Among the inhibitory protein factors, chondroitin sulfate proteoglycans (CSPGs) are abundantly expressed by reactive astrocytes and other cells in scar tissue, which are considered major inhibitors of axonal regeneration during this period. Jerry Silver laboratory showed that reactive astrocyte-derived CSPGs in scar tissue after SCI inhibited the growth of axons in vitro, suggesting a possible inhibitory role in vivo [12]. Later, Bradbury et al. showed that injection of chondroitinase ABC, a CSPG-degrading enzyme, into the lesion could inactivate the CSPGs in the scar tissue, which can effectively induce an improvement of motor function [112]. Furthermore, the thermostabilized chABCs have a more effective influence on axonal sprouting and functional recovery after SCI [113].The results from the Jerry Silver laboratory also showed that axonal growth is prevented when protein tyrosine phosphatase (PTP*σ*) in axons interact with CSPGs in glial scars. They generated a membrane-forming peptide mimetic of the PTP*σ* wedge domain that binds to PTP*σ* and relieves CSPG-mediated inhibition, improving axonal recovery in mice with SCI [114]. Other studies have suggested that inhibiting the formation of CSPGs results in recovery of spinal cord function [115, 116].

#### 3.2.2. Impact of Reactive Astrocytes on Differentiation of Oligodendrocyte Precursor Cells (OPCs)

Oligodendrocyte precursor cells (OPCs) are the most proliferative and widely distributed precursor cells in the CNS, which can supplement the large number of oligodendrocytes lost after SCI. Experimental spinal cord injury in rodents proves that oligodendrocyte precursor cells have extremely powerful remyelination ability [84]. Siebert et al. found that CSPGs, particularly when generated following SCI, inhibit OPC process outgrowth and differentiation, which can be completely reversed by chABCs. Therefore, chABC treatment may not only enhance regenerative axonal sprouting but may also enhance remyelination after SCI [117, 118]. In addition to CSPGs, the expression of bone morphogenetic proteins (BMP) is dramatically elevated in reactive astrocytes. Wang et al. found that upregulation of BMP in reactive astrocytes is the main factor that inhibits the differentiation of OPCs. They suggested that the manipulation of BMP signals in endogenous or grafted OPCs may be a useful therapeutic strategy for increasing OPC differentiation and remyelination after SCI [119]. The expression of endothelin-1 (ET-1), which is a secreted signal peptide, can be upregulated in reactive astrocytes. Further, ET-1 can promote the generation of reactive astrocytes in demyelinating tissue [120]. Hammond et al. showed that ET-1 from reactive astrocytes inhibited the differentiation and remyelination of OPCs by activating the Notch pathway [121]. Thus, ET-1 inhibitor maybe a promising target for controlling reactive astrogliosis and improving SCI recovery.

### 3.3. Positive Function of Reactive Astrocytes in SCI

Of course, in addition to the negative effects of the abovementioned reactive astrogliosis in SCI, there are many positive effects of reactive astrocytes that have been proven or considered.

Contrary to what occurs in the chronic state, reactive astrocytes in the acute phase migrate to the lesion and polymerize inflammatory cells to limit the extent of inflammation, a process that plays an important role in the repair of injury. Okada et al. found that astrocytes migrate to the SCI lesion core and limit the infiltrating inflammatory cells. This indicates that, in the acute phase of SCI, astrocytes contribute to the repair of damage and the recovery of motor function [10]. Similarly, selective reactive astrocyte ablation after SCI causes severe leukocyte infiltration, tissue disruption, demyelination, and neuronal death in GFAP-TK transgenic mice [122]. After SCI, the nerve at the damaged tissue is exposed to a toxic microenvironment, characterized by an imbalance of ionic, amino acids and high levels of inflammatory substances. This environment has a considerable negative impact on the recovery of the spinal cord. Therefore, the scar tissue formed by reactive astrocytes can block the harmful microenvironment [123]. In the early stage of SCI, glutamate was also released nonspecifically into the extracellular environment. Astrocytes were reported to specifically absorb glutamate, thus reducing excitotoxic damage [124]. In addition, glial scars and extracellular matrices stimulate and recruit fibroblasts and endothelial cells, thus also helping to form microvessels at the lesion at a rate which is 50 times higher than that of uninjured areas. Astrocytes have a direct effect on immune cells by secreting immunomodulatory molecules such as TGF-*β*, TNF-*α*, and proteoglycans. CSPGs are peripheral components associated with immune activity. They can adhere to growth factors and chemokines required for immune cell growth and recruitment, thereby increasing the concentration of immune cells. Glial scars may also be involved in the proliferation of neural stem cells and progenitor cells. In relation to this, astrocytes are involved in the proliferation of neural progenitor cells and in the regulation of stem cell proliferation and differentiation. CSPGs play a role in the maturation and growth of neural stem cells. Experiments have shown that injection of chABCs into the telencephalic ventricle resulted in a decrease in the number of ventricular cells and self-renewing glial cells.

As mentioned above, the function of reactive astrocytes varies greatly in different situations. Thus, the pros and cons of reactive astrocytes should be analyzed from a dialectical perspective. Different roles of RA in SCI and other CNS disorders are summarized in Table 3.

### 3.4. Disruption of the Function of Reactive Astrogliosis in SCI

Anderson et al.'s team used three genetically targeted loss-of-function manipulations in adult mice and showed that preventing astrocyte scar formation, attenuating scar-forming astrocytes, or deleting astrocyte scars all failed to result in spontaneous regrowth of transected corticospinal tract or sensory recovery in severe SCI. Attenuating scar-forming astrocyte formation indeed hindered the formation of scars, but it cannot cause effective axons to reenter the injured area. Moreover, scar-forming astrocytes have no significant effect on the deposition of CSPGs. Through RNA sequencing, the authors revealed that astrocytes in SCI lesions express multiple axon-growth-supporting molecules. Therefore, they believe that, contrary to the traditional concept, astrocyte scar formation aids rather than prevents CNS axon regeneration after SCI [140]. Later, however, Silver issued a review stating that the statement of Anderson et al.'s team is untenable [141]. Silver pointed out that Anderson et al. stated that reactive astrocytes form the wall containing proteoglycan, thus blocking the regeneration of axons, which is the only reason for the failure of axon regeneration. In turn, blocking this inhibitory effect will cause spontaneous regeneration of axons. There are other potent regeneration-blocking cell types that are present within the lesion core and penumbra. For example, it neglects the immune compartment as well as the cascade of deleterious effects of lesion-derived and damage-induced microglial activation, which may also lead to regeneration [140]. The hypothesis that only removal of astrocytes inevitably leads to spontaneous regeneration is oversimplistic, especially in such an obviously inflammatory environment. The glial scar composition is complex, and simply changing a certain type of cell to influence the repair process may result in an inaccurate representation. The composition of the scar is also a dynamic process that changes over time. As mentioned earlier, scarring composition promotes repair and regeneration during the early stages of formation, but it hinders regeneration in the chronic phase of SCI [142–144]. Many specific aspects of astrocyte polarization have not been clearly confirmed, such as the number of specific polarized cell types, as well as the type of mediator pathway involved in each type and its role when in contact with surrounding inflammatory cells. Understanding these will be of great help in the development of SCI treatment. Importantly, a consideration of the role of reactive astrocytes in SCI should include a comprehensive and correct understanding of their benefits and disadvantages.

## 4. Therapy Related to Reactive Astrocytes in SCI

**Figure 3:**
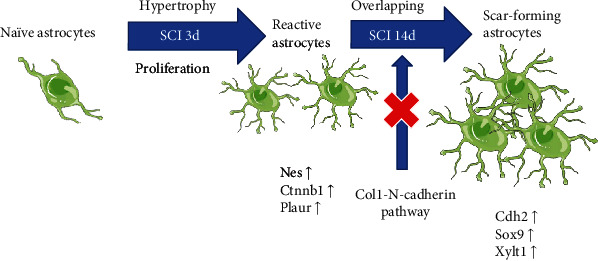
Based on the morphology and function at different stages, astrocytes in SCI are divided into naïve astrocytes, reactive astrocytes, and scar-forming astrocytes. This is a promising method to inhibit the formation of SAs through blockage of the Col1-N-cadherin pathway before the chronic stage of SCI. Nes: nestin; Cdh2: cadherin 2; Sox9: SRY-box transcription factor 9; Xylt1: xylosyltransferase 1; Ctnnb1: catenin beta 1; Plaur: plasminogen activator, urokinase receptor.

In 2017, Hara et al. used laser microdissection in combination with immunohistochemistry in a mouse model of contusion SCI to isolate each astrocyte cell type, including naive astrocytes (NAs), reactive astrocytes (RAs), and scar-forming astrocytes (SAs); as a result, they identified some cell-specific genes. We have listed some characteristics of these cell types in Table 4. In their study, they isolated GFP-positive NAs from primary astrocyte cultures derived from transgenic (CAG-EGFP) mice that ubiquitously express EGFP and transplanted them into the spinal cords of either naive mice or mice with SCI. They then examined the morphological changes of these cells and gene expression. As a result, NAs in the normal spinal cord did not change in either case; however, NAs at the injured spinal cord were polarized. These findings indicate that astrocytes change their phenotype in an environment-dependent manner. Through genetic analysis, Hara et al. also found that genes associated with the extracellular matrix play an important role in the formation of SAs, especially type I collagen (Co1I) genes, which are expressed in the injured spinal cord but not in the normal spinal cord. These findings suggest that type I collagen genes are directly involved in the transformation of RAs into SAs, as well as in astrocytic scar formation, after SCI. This may indicate that the scar formation in the chronic phase can be reduced and regeneration can be promoted by inhibiting the expression of the *Col I* genes. Moreover, Hara et al. showed that Col I genes enhance N-cadherin-mediated contacts in RAs and induce the transformation of these cells into SAs. Type I collagen induces the transformation of RAs into SAs via the integrin–N-cadherin pathway. The authors used anti-*β*1 antibody and collagen-binding integrin to selectively block the formation of scar-forming astrocytes on days 9 to 13 of the chronic phase and to attenuate the transformation of RAs into SAs. The inhibition of the integrin-mediated RA–Col I interaction prevents astrocytic scar formation via N-cadherin downregulation and promotes axonal regeneration and functional recovery after SCI [142]. In order to express the meaning of this more clearly, we have shown the above process in Figure 3. Polymer nanoparticles (NPs) have a wide range of uses in terms of size, potential surface, and hydrophilic or lipophilic properties and have considerable advantages in drug delivery, which can improve drug selectivity and control long-term drug release. Rolipram is an anti-inflammatory drug that acts on the NF*κ*B pathway in astrocytes. In 2020, Vismara et al. found that rolipram-loaded nanogel (NG) could limit expression of proinflammatory substances, including Lcn2 and iNOS, in A1 astrocytes [145]. Furthermore, NG improved motor functional recovery and reduced reactive astrogliosis in the early stage after SCI. This experiment provides a new insight to future therapeutic treatment of SCI. In a previous section of this review, we mentioned that TGF-*β* has the ability to prevent astrocyte phenotypic transformation. In vitro use of recombinant human TGF-*β*3 can rapidly transform A1 into a nonreactive state. Furthermore, recombinant human TGF-*β*3 has been used in clinical trials to promote skin wound healing and scar healing [146]. Similarly, our team revealed the function of miR-21 in astrocyte activity [65, 66] and found that the formation of A1 astrocytes, as well as increments in the production of A2 astrocytes, can be triggered by blocking the miR-21 pathway. Another factor that can be used for the treatment of spinal cord injury is glial-cell-derived neurotrophic factor (GDNF) which, when combined with transplanted Schwann cells, can effectively alleviate the negative effects of RAs after injury. It has been observed that astrocytes migrate to the lesion in large quantities, and the migrated astrocytes are closely related to axons. The processes of astrocyte have been shown to be accompanied by axonal elongation, suggesting that they have a role in repair processes. Notably, GDNF treatment can reduce the production of GFAP and CSPGs [147]. Taken together, the above studies demonstrate that the formation of scar-forming astrocytes can be prevented through a specific pathway before the chronic phase, thereby improving nerve regeneration.

## 5. Problems and Prospects

We have noticed various aspects of reactive astrogliosis in a broad range of contexts, from SCI and neurodegenerative diseases to tumor, even if the overall understanding of reactive astrocytes is still at an early stage. Importantly, the function of astrogliosis is critical for health and disease. Illustrating the underlying mechanisms involved may shed light on possible cures for a wide range of diseases. Currently, there is a lack of clearly specific markers to distinguish between different types of RAs. In earlier research, various typical markers have been raised, but they have only been useful in a limited number of species. It is unclear whether there are other types or mixed types. Additionally, an in vivo model that integrates the pathological process of reactive astrogliosis in a coherent way is lacking. The existing inflammatory and ischemic models are artificial, and they may neglect some important interactions that occur in vivo. At present, there are several limitations in our understanding of the types of reactive astrocytes, and many questions are still unanswered. What types of astrocytes exist in addition to A1 and A2? Do A1 and A2 astrocytes represent opposite poles; that is, is one beneficial while the other is harmful? Are there intermediate forms between these two? Is the fate of reactive astrocytes related to the difference in their intrinsic genes? The mechanism of A1 formation has been roughly elucidated, but the induction pathway for the formation of A2 astrocytes remains largely unknown. How do reactive astrocytes differentiate into scar-forming astrocytes in SCI? In the face of related issues, single-cell sequencing technology and spatial transcriptomics technology can provide us with partial answers. We can use the fluorescent-activated cell-sorting technique to select specific polarized astrocyte populations for single-cell sequencing and perform spatial transcriptome by slicing different levels of the spinal cord after injury. Compared with traditional sequencing, single-cell sequencing analyzes genes within specific single cell rather than cell populations. The pseudosequence single-cell sequencing technology allows us to understand the various types of polarized cells after SCI and to determine their specific genotypes, as well as to discover more specific markers of polarized cells. Further, we can monitor the dynamic changes of cells like A1 and A2. This will perhaps help us understand how naïve astrocytes transform into scar-forming astrocytes. Further research is needed on the role of reactive astrogliosis in SCI. Are the RAs formed in the acute phase as helpful in SCI recovery as described above? What are the side effects? By the same token, are there only absolute side effects of SAs formed in the chronic phase? What are the adverse effects of blocking it? These questions will drive the direction of future research. There is still a long way to go in this intriguing area of astrocyte reactivity. Fortunately, with the development of technology, more precise separation and purification instruments have emerged. Moreover, microscopy is also rapidly progressing, and we can more clearly observe the specific details of reactive astrocytes, which is of great value for research. We believe that more researchers will join the study of astrocyte polarization, which will greatly promote the progress of this field. The solution to these problems will bring hope to patients with SCI.

## 6. Conclusions

In this review, we briefly described the roles of astrocytes and the concept and characteristics of astrocyte polarization. Further, we described the roles of astrocytes in SCI and the pathological changes involved. Finally, we explored the implication of astrocytes in the treatment of SCI. The existing evidence supports the hypothesis that scar formation can be reduced by blocking certain pathways, which provides a new direction for future SCI treatment. We also discussed some existing problems in this field and proposed possible solutions. Last but not least, we provided a picture of the future development and prospects in the field of reactive astrogliosis.

## Figures and Tables

**Table 1:** Signaling pathways and molecules implicated in formation of reactive astrocytes.

Categories	Molecules
Extracellular molecules and signaling pathways	
Cytokines & growth factors	IL-1*β*, IL-1*α*, IL-6, IL-10, IL-17, TNF-*α*, IFN-*γ*, CNTF, EGF, Sonic hedgehog (SHH) [32, 70, 71]
Neurodegeneration-associated molecules	Amyloid-beta, *α*-synuclein [6]
Transmitters	Glutamate, noradrenalin [72]
Oxidative stress molecules	NO, ROS [73]
Immune-related molecules	LPS, Toll-like receptor ligands [74]
Hormones	Estrogens, glucocorticoids [75]
Intrinsic molecules in reactive astrocytes	
Signal transducers	STAT3, NF*κ*B, JAK2, MAPK, SOX9, mTOR, c-JUN, Olig2, SOC3, PKA, PKC [31, 57]
Receptors	EGFR, cannabinoid-2 receptor (CB2R), CD36 [76, 77]
MicroRNAs	miR-21, miR-140, miR-145, miR-17 [65, 78–80]

CNTF: ciliary neurotrophic factor; EGF: epidermal growth factor; ROS: reactive oxygen species; PKA: protein kinase A; PKC: protein kinase C.

**Table 2:** Molecules and gene expression of reactive astrocytes.

Categories	Intercellular molecules
Cytokines	IL-1*β*, IL-6, IL-10, TNF-*α*, INF-*γ*, TGF-*α*, TGF-*β*, CNTF, LIF, CLCF1 [26, 63, 64, 85, 86]
Chemokines	CCL2, CCL3, CCL4, CCL5, CXCL1, CXCL2, CXCL10, CCL12, CXCL20 [87, 88]
Amino acids & glutathione	GABA, glutamate, d-serine [52, 89–92]
Oxidative stress substances	ROS, NO, NOS [92–94]
Extracellular matrix	CSPGs, connective tissue growth factor, collagen I, fibronectin, MMP-9 [12, 95, 96]
Growth factors	VEGF, FGF-2, BDNF, GDNF [71, 97–99]
Categories	Intracellular molecules
Transcriptional regulators	STAT3, NF*κ*B, Olig2, SOX9, mTOR [60, 62, 63, 100, 101]
Receptors & ion channels	EGFR, KCa3.1, AQP4 [67, 102, 103]
Intermediate filaments	Nestin, vimentin, GFAP [16, 88]

BDNF: brain-derived neurotrophic factor; GDNF: glial cell line-derived neurotrophic factor; VEGF: vascular endothelial growth factor; EGFR: epidermal growth factor receptor; MMP-9: matrix metalloproteinase-9; GABA: gamma-aminobutyric acid; CLCF1: cardiotrophin-like cytokine.

**Table 3:** Positive and negative influence of reactive astrogliosis.

*Positive effects*	*References*
Limits leukocyte infiltration, repairs the blood–brain barrier	[122, 125–127]
Uptakes excess glutamate and prevents chronic glutamate neurotoxicity	[124, 128]
Promotes neuronal survival as well as axonal regeneration	[28, 129, 130]
Alleviates neurodegenerative disease	[131, 132]
Maintains the brain's excitation/inhibition balance, prevents the development of epileptic seizures	[133]

*Negative effects*	*References*
Limits axonal regeneration and functional recovery after SCI	[112, 134–136]
Contributes to the development and persistence of chronic pain	[137, 138]
Inhibits differentiation of oligodendrocyte precursor cells	[117–120]
Fosters brain metastases via STAT3 signaling	[139]

**Table 4:** Some specific characteristics of reactive astrocytes and scar-forming astrocytes.

	Reactive astrocytes	Scar-forming astrocytes
Marker gene	Nes, Ctnnb1, Plaur, Mmp2, Mmp13, Axin2	Cdh2, Sox9, Xylt1, Chst11, Csgalnact1, Acan, Pcan, Slit2
Function	(1) Seclude inflammatory cells (2) Lead to tissue repair and functional improvement	(1) Form astrocytic scar (2) Impede CNS axonal regeneration resulting in limited functional recovery
Emergence time	3 d–14 d	14 d
References	[10]	[14, 148]

Mmp2: matrix metallopeptidase 2; Chst11: carbohydrate sulfotransferase 11; Csgalnact1: chondroitin sulfate N-acetylgalactosaminyltransferase 1; Acan: aggrecan; Slit2: slit guidance ligand 2.

## Abbreviations

CNS: Central nervous system
GFAP: Glial fibrillary acidic protein
LPS: Lipopolysaccharide
NAs: Naïve astrocytes
SCI: Spinal cord injury
TGF-*β*: Transforming growth factor *β*
CSPGs: Chondroitin sulfate proteoglycans
ChABCs: Chondroitin sulfate proteoglycans
PTP*σ*: Protein tyrosine phosphatase *σ*
OPCs: Oligodendrocyte precursor cells
BMPs: Bone morphogenetic proteins
RAs: Reactive astrocytes
SAs: Scar-forming astrocytes.
